# Floral gene resources from basal angiosperms for comparative genomics research

**DOI:** 10.1186/1471-2229-5-5

**Published:** 2005-03-30

**Authors:** Victor A Albert, Douglas E Soltis, John E Carlson, William G Farmerie, P Kerr Wall, Daniel C Ilut, Teri M Solow, Lukas A Mueller, Lena L Landherr, Yi Hu, Matyas Buzgo, Sangtae Kim, Mi-Jeong Yoo, Michael W Frohlich, Rafael Perl-Treves, Scott E Schlarbaum, Barbara J Bliss, Xiaohong Zhang, Steven D Tanksley, David G Oppenheimer, Pamela S Soltis, Hong Ma, Claude W dePamphilis, James H Leebens-Mack

**Affiliations:** 1 Natural History Museum, University of Oslo, NO-0318 Oslo, Norway; 2 Department of Botany, University of Florida, Gainesville, FL 32611, USA; 3 School of Forest Resources, The Pennsylvania State University, University Park, PA 16802, USA; 4 Interdisciplinary Center for Biotechnology Research, University of Florida, Gainesville, FL 32610, USA; 5 Department of Biology and Huck Institutes of the Life Sciences, The Pennsylvania State University, University Park, PA 16802, USA; 6 Department of Plant Breeding, Cornell University, Ithaca, NY 14853, USA; 7 Department of Botany, Natural History Museum, London SW7 5BD, United Kingdom; 8 Faculty of Life Sciences, Bar-Ilan University, Ramat-Gan 52900, Israel; 9 Department of Forestry, Wildlife and Fisheries, University of Tennessee, Knoxville, TN 37996, USA; 10 Florida Museum of Natural History, University of Florida, Gainesville, FL 32611, USA

## Abstract

**Background:**

The Floral Genome Project was initiated to bridge the genomic gap between the most broadly studied plant model systems. *Arabidopsis *and rice, although now completely sequenced and under intensive comparative genomic investigation, are separated by at least 125 million years of evolutionary time, and cannot in isolation provide a comprehensive perspective on structural and functional aspects of flowering plant genome dynamics. Here we discuss new genomic resources available to the scientific community, comprising cDNA libraries and Expressed Sequence Tag (EST) sequences for a suite of phylogenetically basal angiosperms specifically selected to bridge the evolutionary gaps between model plants and provide insights into gene content and genome structure in the earliest flowering plants.

**Results:**

Random sequencing of cDNAs from representatives of phylogenetically important eudicot, non-grass monocot, and gymnosperm lineages has so far (as of 12/1/04) generated 70,514 ESTs and 48,170 assembled unigenes. Efficient sorting of EST sequences into putative gene families based on whole *Arabidopsis*/rice proteome comparison has permitted ready identification of cDNA clones for finished sequencing. Preliminarily, (i) proportions of functional categories among sequenced floral genes seem representative of the entire *Arabidopsis *transcriptome, (ii) many known floral gene homologues have been captured, and (iii) phylogenetic analyses of ESTs are providing new insights into the process of gene family evolution in relation to the origin and diversification of the angiosperms.

**Conclusion:**

Initial comparisons illustrate the utility of the EST data sets toward discovery of the basic floral transcriptome. These first findings also afford the opportunity to address a number of conspicuous evolutionary genomic questions, including reproductive organ transcriptome overlap between angiosperms and gymnosperms, genome-wide duplication history, lineage-specific gene duplication and functional divergence, and analyses of adaptive molecular evolution. Since not all genes in the floral transcriptome will be associated with flowering, these EST resources will also be of interest to plant scientists working on other functions, such as photosynthesis, signal transduction, and metabolic pathways.

## Background

The genome sequences of *Arabidopsis *[1] and rice [2,3] have stimulated great advances throughout the plant sciences. Comparisons of these eudicot and monocot genomes have provided many insights into the genome characteristics and evolutionary histories of both lineages [e.g. [4-6]], and comparisons involving additional species are generating a more global picture of angiosperm genome evolution [7-9].

**Figure 1:**
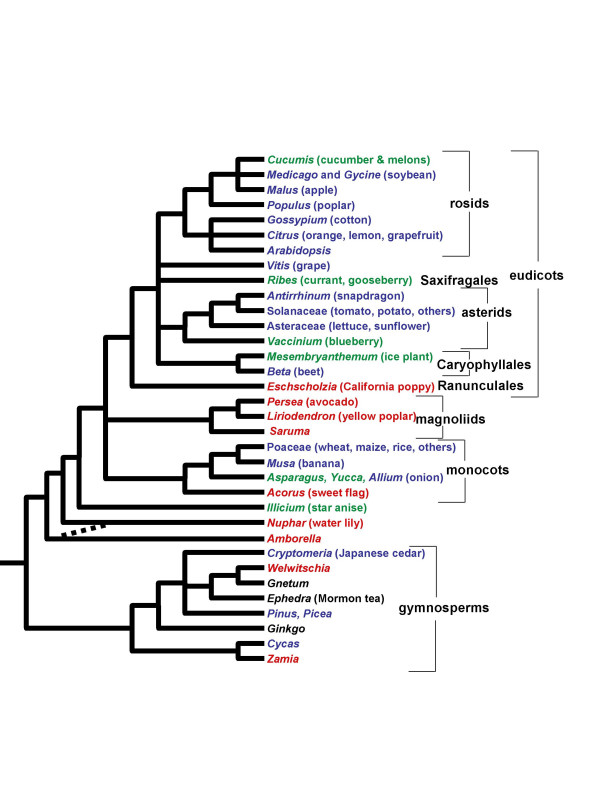
Well- supported evolutionary relationships among FGP species and other genomic models are shown in this phylogenic tree of seed plants. Red taxon names indicate those species for which we aim to sequence 10,000 ESTs, green taxon names indicate species for which we are sequencing 2000 ESTs, and blue taxon names indicate species for which large EST sets are already available in public databases [24, 25, 28, 29, 61] or will soon become available [70].

These multispecies comparisons, and comparative plant sciences more generally, have been aided by the well-supported understanding of evolutionary relationships among flowering plants that has emerged over the last decade [e.g. [10-13]]. Among the most noteworthy phylogenetic results is the well-supported inference that whereas monocots form a clade, the dicots as traditionally circumscribed do not. Rather, monocots are derived from within the "primitive" dicot grade, now collectively referred to as basal angiosperms (Fig. 1). The "eudicots" (or "tricolpates" [14]; Fig. 1) do form a clade that comprises ca. 75% of all angiosperm species [15], and most of this diversity is found among the "core" eudicots, which include the rosids, asterids, and Caryophyllales (Fig. 1). Model systems such as *Arabidopsis thaliana*, tomato (*Lycopersicon esculentum*), cotton (*Gossypium*), poplar (*Populus*), barrel medic (*Medicago truncatula*) and ice-plant (*Mesembryanthemum crystallinum*), are all representatives of the core eudicot clade (Fig. 1). Rice (*Oryza savita*), maize (*Zea mays*), wheat (*Triticum aestivum*), barley (*Hordeum vulgare*), sorghum (*Sorghum bicolor*), and sugarcane (*Saccharum officinale*) are all members of the grass family (Poaceae), a phylogenetically derived lineage within the monocots (Fig. 1). Although comparisons of the rice and *Arabidopsis *genomes will undoubtedly identify many features of the ancestral angiosperm genome, this pair-wise comparison alone will not be able to distinguish *Arabidopsis*-specific attributes from those specifically absent in rice or visa versa. The recent posting of high coverage genome sequence for *Populus trichocarpa *[16] is a major advance for comparative plant genomics, but even *Populus*-*Arabidopsis*-rice comparisons cannot distinguish features common to all angiosperms from those that arose in the most recent common ancestor of eudicots and monocots, which existed at least 125 million years ago [17] and perhaps more than 140 million years ago [e.g. [18-20]]. In general, the resolving power of comparative plant genomics will increase with additional taxa representing key lineages in plant phylogeny (Fig. 1). Increased genomic resources for phylogenetically diverse plant species will lead to a better understanding of plant genome evolution, the diversification of gene families, and the origins of reproductive characteristics common to all flowering plants.

Basal angiosperms and basal eudicots (e.g., Ranunculales), while comprising a small percentage of the total number of extant angiosperm species, nonetheless encompass an astonishing spectrum of developmental patterns and floral forms [21,22]. In turn, this diversity provides a clear opportunity to reconstruct to the basal condition of angiosperms, and thereby bridge the evolutionary gap between model eudicot and monocot genomes. Understanding the evolution of angiosperm genes and genomes, including the floral transcriptome, requires three-way and higher-order comparisons that extend beyond *Arabidopsis *and rice. This point is widely appreciated, and comparative plant genomics is being fueled by the availability of genomic resources for a growing number of plant species. The addition of species representing basal angiosperms, basal eudicot, and non-grass monocot lineages will be especially valuable, not only for flowering research, but also for more general "reconstructomic" studies of housekeeping and transcription factor functions.

A primary objective of the Floral Genome Project (FGP; [23]) is to uncover patterns of conservation and divergence of the floral transcriptome among angiosperms, particularly to elucidate the role of gene duplications and shifting expression patterns in the origin and diversification of angiosperms. The FGP has constructed a large collection of non-normalized nor tissue subtracted cDNA libraries and 5' EST sets from developing reproductive tissues for selected species of basal eudicots, basal angiosperms and gymnosperms (Table 1). These species represent not only key nodes in the angiosperm phylogenetic tree and its sister group (gymnosperms), but also a diversity of reproductive structures and developmental patterns. While multi-species comparisons of large sequence data sets are already possible for Poaceae [9,24] Solanaceae [25], and Brassicaceae [7,26,27], the addition of large EST sets for basal angiosperms opens the door to fundamental comparative genomics investigations of the origin and diversification of flowering plants.

## Results

Random 5' sequencing of cDNAs from basal flowering plants has so far (as of 12/01/04) generated 70,514 ESTs assembled into 48,170 unique gene sequences (Table 1). These materials should provide essential resources for comparative genomic research because they represent previously poorly sampled genomes placed at crucial points in angiosperm phylogeny. Gene sequences from the gymnosperm *Welwitschia*, the basalmost angiosperms *Amborella *and *Nuphar*, the basal monocot *Acorus*, the magnoliids *Persea*, *Liriodendron *and *Saruma*, and the basal eudicot *Eschscholzia *will aid in placing boundary dates on the origins of florally-expressed gene families, help resolve patterns of gene and genome evolution within the flowering plants, and bridge critical gaps in comparative analyses involving monocot and eudicot model systems. Identification of cDNA clones for finished sequencing has been aided by efficient sorting of EST sequences into putative gene families based on whole *Arabidopsis*/rice proteome comparison. Phylogenetic analyses of ESTs are providing new insights into the process of gene family evolution in relation to the origin and diversification of the angiosperms. Here we introduce our EST database and provide some examples of broad utility of these data in comparative analyses.

### PGN website

All FGP EST data and unigene builds are available through the Plant Genome Network (PGN) website [28], linked also through the FGP homepage [29]. PGN was designed as a general-purpose EST analysis pipeline and web-based database that can be readily employed as a "front end" for other EST sequencing projects. PGN is a trace file database accepting all standard automated sequencer file formats. Quality information in the raw trace files is used for sequence trimming and assembly, and chromatograms can be visualized through the website. The focus on trace file data distinguishes PGN from other EST databases such as PlantGDB [30] and the TIGR Gene Indices [31]. PGN also provides an EST processing and annotation service for smaller EST projects that may not have the informatics resources to generate a public database, and provides a stable web address for these projects. PGN provides public access to EST library statistics, unigene build details, EST chromatograms, and permits FGP taxon-specific BLAST [32] searches.

### Tribe analysis

Tentative classification of unigenes has allowed us to identify quickly the genes represented in our EST sets. We created an objectively defined scaffold for classification through cluster analysis of the *Arabidopsis *and rice proteomes. The PlantTribes database [33] can be searched using BLAST, or by query with *Arabidopsis *or rice sequence IDs [34], sequence annotations, Pfam accession IDs [35] or keywords.

To construct PlantTribes, predicted protein sequences from the *Arabidopsis thaliana *var. Columbia and *Oryza sativa *var. *japonica *(rice) genomes were downloaded from TIGR [34]. The BLASTP program [32] was used to compare all sequences to each other, and the similarity-based clustering procedure TribeMCL [36,37] was used to group proteins into putative gene families within our PlantTribes database. Of the 20,992 tribes identified by MCL cluster analysis of the *Arabidopsis *and rice proteomes, 60 PlantTribes included at least one of 100 known floral development regulators (Tables 2 and 3).

### Unigene overlap

**Figure 2:**
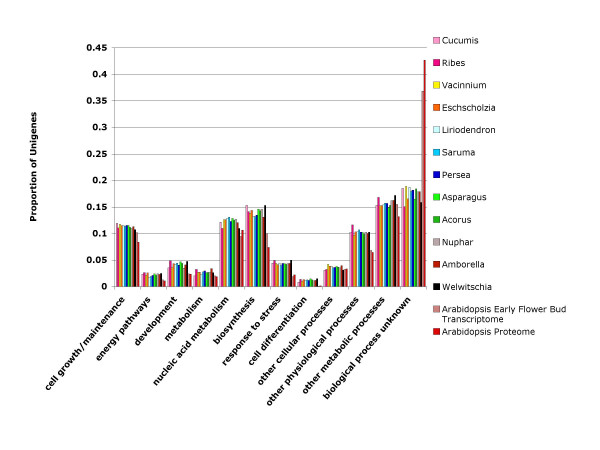
The relative frequencies of ESTs assigned to GO Biological process classes are quite similar across our study taxa. Class frequencies are shown for ten EST sets, the inferred Arabidopsis proteome, and Arabidopsis genes with moderate-to-high expression in young inflorescences (stage 3).

To estimate the complexity of the non-normalized nor tissue subtracted FGP cDNA libraries and the underlying floral transcriptomes, we analyzed predicted functions of the FGP unigenes. Overall, functional classification of FGP unigene assemblies shows that EST sequencing has captured a nearly uniform representation of the sampled transcriptomes. On average, 53% of unigenes from each taxon match *Arabidopsis *genes with an e-value of 1.0e^-10 ^or better. An analysis of GO annotations [38] for these genes shows that the FGP unigene sets provide a remarkably consistent sampling of functional classes defined for the *Arabidopsis *proteome (Fig. 2). Moreover, similar GO classification frequencies were observed in a subset of 11,974 genes that were found to be expressed at moderate-to-high levels (>100 units) in an Affymetrics microarray analysis of young (stage 3) *Arabidopsis *inflorescences (Zhang et al. unpublished data).

**Figure 3:**
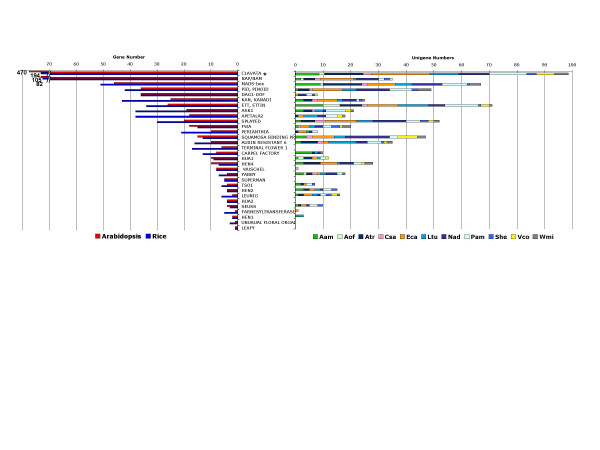
TribeMCL gene clusters (Tribes) with floral development genes vary in size and tend to include similar numbers of rice and *Arabidopsis *genes (left). These gene families are well represented in our EST sets (right and Tables 2 and 3). *The unigene counts (right) for the *CLAVATA *gene family have been halved.

Estimation of unigene overlap is inherently error-prone because best BLAST hits are not necessarily orthologs. Moreover, even when orthology is established through formal phylogenetic analysis, similarity in function does not necessarily follow orthology [e.g. [39]]. We used the PlantTribes database to estimate the overlap among our unigene sets at the gene family level. Unigenes were sorted into the tribes if they have best BLASTX hits to any member of the tribe. Each taxon has unigenes sorted to 19–51% of the 60 tribes that include floral development regulators (Tables 2 and 3). On average, 70% of the gene families represented in one EST set are represented in at least one other EST set. As expected, the most overlap occurs in the largest gene families (Fig. 3).

Among the 100 *Arabidopsis *floral regulatory genes identified from the literature (Tables 2 and 3), 67 have closely related homologs (best BLAST hit) in at least one FGP EST set. On average, ESTs with a best hit among the 100 listed *Arabidopsis *flower development genes constitute approximately 1% of each FGP EST set. The average overlap of best hits to these floral regulatory genes was 30.8% between pairs of EST sets. The *Amborella*, *Nuphar *and *Eschscholzia *unigene sets shared three-way overlap in best BLAST hits to six floral development genes (*AGAMOUS*, *AGL6*, *APETALA3*, *SEPALLATA1*, *AXR6*, and *YABBY3*; Table 2) and these species plus *Persea *shared four-way overlap in best BLAST hits two of these genes (*APETALA3 *and *AXR6*). In addition, the FGP ESTs/unigenes were found to match on average 4.5% of the apparent single-gene/taxon tribes (representing putative single-copy genes) from *Arabidopsis*. The average overlap of these putative single copy genes between two FGP taxa was 28%, and three-way overlap of such genes among the *Amborella*, *Nuphar *and *Eschscholzia *EST sets was 10% (representing 26 genes).

The high frequency (66%) of crucial *Arabidopsis *floral regulators identified in BLAST searches as best hits for sequences in one or more of our EST sets (Tables 2 and 3) indicates that FGP EST sets are a valuable resource for comparative floral developmental studies. For example, identifying homologs of genes being investigated in model systems opens the door to broad cross-species comparative analyses of gene function. Of the 1453 *Arabidopsis *genes that have recently been identified as having organ-specific expression within developing flowers [40], 388 (27%) are best BLAST matches to genes from at least one of our unigene sets (e.g. genes in Table 4). We consider this to be a high percentage, given that our cDNA libraries were constructed from a subset of the developmental stages analyzed in the *Arabidopsis *study [40].

Given what is already known about gene duplications in angiosperm history [e.g. [41-43]], the BLAST based measures of overlap are almost certainly underestimates of cross-taxon sampling of orthologous gene sets (including co-orthologs [44]) represented in our EST sets. Whereas the measures of among-taxa overlap in gene families as defined in the PlantTribe database provide a possible upper bound on the degree of overlap among orthologous sets, simple comparison of best BLAST hits in the *Arabidopsis *proteome provides a likely lower bound.

Formal phylogenetic analyses provide a more accurate assessment of orthologous gene sets. For example, within the MIKC MADS-box gene family, phylogenies uncover greater levels of overlap among our EST sets than were inferred from simple BLAST-based analyses. Of the 12 taxa listed in Table 1, representatives of the *DEFICIENS*, *GLOBOSA*, *AGAMOUS*, *FRUITFULL/SQUAMOSA*, *SEPALLATA*, *AGL6*, and *TM8 *clades have been identified in 8, 6, 5, 3, 7, 9 and 2 unigene sets, respectively.

In addition to providing more accurate estimation of overlap among the transcriptomes being sampled in EST studies, phylogenetic analyses of gene families provide insights into the evolutionary history of genes characterized in model systems. For example, recent phylogenetic surveys of MADS box genes have identified gene duplication events that appear to be associated with the origin and rise of angiosperms and the radiation of core eudicots [e.g., [45-50]].

**Figure 4:**
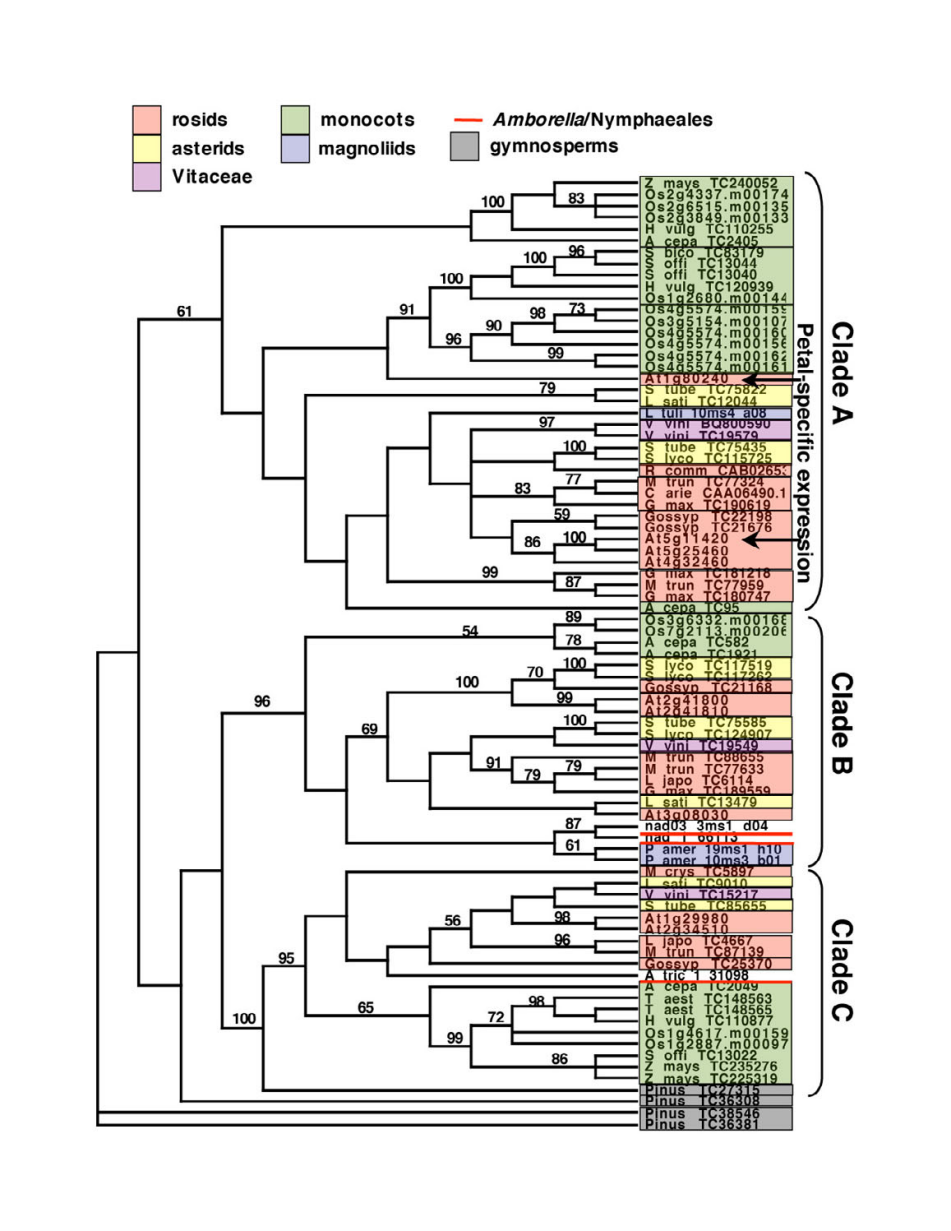
A phylogeny for the DUF642-domain gene family indicates that two *Arabidopsis *genes with differential expression in petals [40] are not the products of a recent duplication event. Genes with the plant-specific but functionally uncharacterized domain family DUF642 form three clades (A, B, and C). In addition to the *Arabidopsis *genes with petal-specific expression patterns, Clade A includes asterid, *Vitis*, monocot and magnoliid genes, indicating that the clade predates the divergence of these lineages (Fig. 1). The genomes of basal-most angiosperm lineages (*Amborella *and Nymphaeales) and gymnosperms may also contain unsampled Clade A genes. Clade B genes were sampled from *Nuphar *(Nymphaeales) and Clade C genes were identified from *Amborella *and *Pinus*. Bootstrap support values (>50%) are shown above each branch. Abbreviated taxon names associated with some gene sequences: Os, *Oryza sativa *(rice); At, *Arabidopsis thaliana*; Gossyp, *Gossypium *spp. (cotton); G.max, *Glycine max *(soybean); L.japo, *Lotus japonicus*; M.trun, *Medicago truncatula*; V.vini, *Vitis vinifera *(grape); L.sati *Lactuca sativa *(lettuce); S.tube, *Solanum tuberosum *(potato); S.escu, *Solanum esculentum *(tomato); M.crys, *Mesembryanthemum crystallinum *(ice plant); A.cepa, *Allium cepa *(onion); T.aest, *Triticum aestivum *(wheat); H.vulg, *Hordeum vulgare *(barley); S.bico, *Sorghum bicolor*; S.offi *Saccharum officinarum *(sugarcane); Z.mays, *Zea mays *(maize); L.tuli, *Liriodendron tulipifera *(tulip poplar); P.amer, *Persea americana *(avocado); N.adve *Nuphar advena*; A.tric, *Amborella trichopoda*.

Phylogenetic analyses of poorly understood gene families can also provide valuable insights into both function and phylogenetic history. For example, two of the 18 genes identified as differentially expressed in petals by Wellmer et al. [40] belong to a single gene family identified in PlantTribes [33]. This gene family includes 10 *Arabidopsis *genes and 16 rice genes, all containing a plant-specific domain, DUF642 [35], the function of which is unknown. DUF642 homologs were identified in ESTs sampled from *Amborella*, *Nuphar*, *Persea*, *Liriodendron *as well as 16 additional plant species included in the TIGR plant gene indices [31]. A phylogeny of these sequences reveals one weakly supported and two well supported subfamilies (Fig. 4). We will refer to these putative subfamilies as clades A, B, and C, respectively (Fig. 4). The well supported placement of a gymnosperm gene (from pine) as sister to all angiosperm genes in clade C indicates that the origin of this subfamily predated the common ancestor of angiosperms and gymnosperms. Asterid, rosid and monocot genes can be identified in each of the three subfamilies; magnoliid genes are placed in clades A and B; and the basal-most angiosperms (*Amborella *and the Nymphaeales) are represented in both clades B and C. The phylogeny suggests that one of the two genes identified by Wellmer et al. [40] is a recent duplicate, the sister gene of which is not differentially expressed in petals. Determining the expression patterns of other DUF642 genes in *Arabidopsis*, rice, and other plant species and mapping this information onto the phylogeny would be a first step toward understanding their current function and functional evolution.

## Discussion

### Proof-of-concept: EST coverage

Relative to the Gene Ontology (GO) functional classification, the FGP is detecting new, translated sequences with astonishing similarity to frequencies known for the entire *Arabidopsis *transcriptome. These gene discovery frequencies support the preliminary hypothesis that the functional complexity of the floral transcriptome roughly equals that of the global plant transcriptome. This point is supported by a comparison of the predicted *Arabidopsis *proteome and the collection of genes identified as moderate-to-highly expressed in young *Arabidopsis *inflorescences (Fig. 2, Zhang et al. unpublished data). Moreover, these detection rates ensure that FGP sequences will be of great interest to the evolutionary biologists for analyses of gene and genome duplications and selection at the molecular level, as well as to the plant molecular biological community in general. For phylogenetics, FGP unigenes assigned to single-gene PlantTribes (such as *FRIGIDA *and *GIGANTEA*) could be used to develop nuclear markers spanning all angiosperms. Indeed, further comparative functional analyses of such single-copy genes could be used to test whether natural selection culls duplicates from plant genomes following segmental or genome-wide duplication events.

### Proof-of-concept: MADS-box genes

The efficacy of a comparative genomics approach to the discovery of genes involved in floral development is illustrated in an analysis of MIKC-type MADS-box genes. To date, 48 MADS unigenes were identified as likely orthologs to major MADS-box groups, including those of the MIKC-type MADS-box genes that encode well-characterized floral regulators, such as *DEFICIENS*, *GLOBOSA*, and *AGAMOUS*. Further phylogenetic and expression analyses of these newly identified genes from the FGP species promise to yield new insights into the evolution of this gene family critical for flower development. In addition, sequences representing the *TM8 *clade have been identified in our *Amborella *and *Persea *EST sets. The *TM8 *gene, expressed in developing tomato flower buds, was sequenced at an early point in the study of MADS-box gene function [51]. Although a *TM8 *ortholog, *ERAF17*, has been associated with female flower development in cucumber [52], no ortholog of *TM8 *has been identified within the *Arabidopsis *or rice genomes, suggesting that it was lost from the genomes of these species. Identification of *TM8 *orthologs in eudicots suggested that the gene duplication establishing the lineage had at least occurred among ancestral core eudicots [45]. However, phylogenetic placement of *Amborella *and *Persea *genes within this clade pushes its origin back to the oldest node in angiosperm phylogeny at least 130 Mya on the basis of fossil evidence [53,54] and perhaps 145–208 Mya according to molecular estimates [20,55], suggesting that the *TM8*-like genes were part of the basic floral "tool-kit" in the earliest angiosperms. Had these new orthologs not been identified, the limited understanding of the functions of *TM8 *and *ERAF17 *would have rendered them unlikely targets for candidate gene analyses of basal angiosperms.

The high capture rate of known floral development regulators in the MADS-box gene family can be considered a proof-of-concept for the FGP. We expect this frequency to increase for MADS-box and other floral gene families as the size of FGP EST libraries and unigene sets expands.

### Limitations

The FGP EST sets described here include sequences from non-normalized nor tissue-subtracted cDNA libraries. As such, many of the genes captured are expressed across many tissues, which could in large part account for the evenly proportioned GO classifications among FGP taxa and the *Arabidopsis* proteome (Fig. 2). Additionally, our approach to EST collection has limited the discovery of transcripts known to be rare, such as for the *SUPERMAN *gene [56]. Nonetheless, this limitation can be overcome by either targeted screening of our cDNA libraries or rtPCR.

Indeed, primer design for PCR amplification has already been aided by use of alignable sequences observed across multiple FGP EST sets. For example, primer pairs designed from alignments of *Amborella *and *Liriodendron *unigenes with *Arabidopsis GIGANTEA *and rice homologs have been used successfully to amplify unsampled sequences in *Nuphar*, *Acorus*, *Eschscholzia *and *Ribes*.

Although EST sequencing alone will result in incomplete sampling of genes and gene families across taxa, they offer many possibilities for further experimentation and hypothesis testing. For example, EST resources provide the opportunity to derive finished coding sequences that will be more useful for genetic manipulation as well as for comparative bioinformatics. Finished full-length cDNAs can be used as tools for many research endeavors, ranging from promoter isolation to anchoring of shotgun genomic sequences. Whereas incomplete sequences for sampled genes may reduce resolution and accuracy in some phylogenetic analyses, parsimony methods are relatively robust with respect to missing data when taxon sampling is extensive [57].

## Conclusion

ESTs and assembled unigenes collected from placeholders for several critical lineages of basal angiosperms are helping to bridge the genomic gap between the eudicot and monocot model plant systems. Initial findings suggest that the basic floral transcriptome, as collected from non-normalized, non-subtractive cDNA libraries, are similar to the inferred *Arabidopsis *transcriptome in the proportions of its GO functional categories. Moreover, the rates of acquisition of known floral gene homologues among the various basal angiosperm EST sets are high. Finally, in one example of floral gene capture, representation among the ESTs of one lineage of the MADS-box gene family has set the origin of that gene group before the divergence of monocots from basal angiosperms. Together, these results provide strong proofs-of-concept for the Floral Genome Project. We anticipate that these initial findings will afford the opportunity to address a number of conspicuous evolutionary genomic questions, including reproductive organ transcriptome overlap between angiosperms and gymnosperms, genome-wide duplication history, identification of lineage-specific gene duplications and functional divergence, and analyses of adaptive molecular evolution. More generally, plant scientists may use the FGP resources and the comparative method to enhance estimates of sequence/domain conservation as well as hypotheses of function among all of the gene families captured. These resources will also be useful for designing both taxon-specific and universal primer sets for amplification and sequencing of specific genes sampled in one or more of our EST sets.

## Methods

### Sampling rationale and molecular methods

We selected species for analysis (Fig. 1, Table 1) by balancing the following major criteria: (1) phylogenetic position, (2) diversity of floral-organ structure (but absence of highly specialized floral features), (3) direct relevance to crop or economic plants, (4) diploid with a small genome size, (5) availability of inbred lines, when possible, (6) possession of other desirable properties, such as large numbers of flowers per plant, transformability, and having been the focus of prior flower developmental study, (7) non-duplication of ongoing studies of the floral transcriptome – i.e., non-duplication of effort with ongoing studies of model plants (*Arabidopsis*, tomato, maize, rice, alfalfa, soybean, cotton, etc.). *Welwitschia *and *Zamia *are gymnosperms representing the gymnosperm phyla Gnetophyta and Cycadophyta, respectively. Sequence data from these species and a growing number of conifer species are providing insights into the gene family content in the most recent common ancestor of angiosperms and gymnosperms. *Amborella*, *Nuphar *(water lily), and *Illicium *(star anise) represent the most basal clades of extant angiosperms. *Saruma*, *Liriodendron *(tulip poplar), and *Persea *(avocado) represent three different orders of magnoliids. *Persea *is a valuable fruit crop and *Liriodendron *is a transformable timber species. *Acorus *(sweetflag) is sister to all other monocot phylogeny and *Eschscholzia *(California poppy) is a transformable species representing the basal eudicots. *Asparagus*, *Cucumis *(cucumber), *Ribes *(currant, gooseberry), and *Vaccinium *(blueberry) are all crop species holding strategic positions in Angiosperm phylogeny. Finally, *Mesembryanthemum *(ice plant) is a model for the study of Crassulacean Acid Metabolism (CAM) in drought-resistant plants [58] and represents the Caryophyllales clade of core eudicots.

Isolating high quality RNA and building cDNA libraries was particularly challenging for many of the FGP taxa, most of which are non-cultivated. The cDNA libraries from these species were constructed using pre-meiotic immature floral tissues (or reproductive structures in the case of gymnosperms), in order to enrich for early floral regulatory genes important for floral patterning and to prevent heavy representation of the many anther-specific genes that are highly expressed in pollen development. Each library, non-subtractive and non-normalized to be most representative of the floral transcriptome, was made with a signature adaptor-linker sequence to eliminate the possibility of misidentification of clones in the future. Overrepresentation of genes among the libraries was further reduced by sampling mRNA from very young floral buds which have a high diversity of cell types and lack the cells, such as tapetum and pollen, that contain a large number of specific transcripts. Detailed methods used for mRNA extraction, cDNA library construction, and information on library attributes (vector, cloning sites, titer, average insert size, etc.) can be found in Table 1 and on the FGP homepage [29].

### Sequence processing pipeline

A standard sequence analysis pipeline was developed consisting of base calling using PHRED [59], vector and *E. coli *sequence contamination screening, and unigene assembly. In addition, a database was developed that also serves as the back end for the Plant Genome Network website [28]. The analysis pipeline and the sequence database are tightly integrated. The quality screen consisted of trimming low-quality sequence, based on PHRED scores, using a custom algorithm. To extend the high-quality sequence as far as possible given a particular quality threshold, the sequence was scanned and, concomitantly, the difference between the quality score and the quality threshold (termed the "adjusted score") was integrated over the length of the sequence. The high-quality sequence was defined as the region of sequence in which the integration of the adjusted score was maximal; this region can include small regions of lower-scoring nucleotides if they are "compensated" by higher-scoring downstream sequence. Next, putative polyA tails were removed if they contained more than 20 consecutive adenine residues. A contamination screen was performed to remove *E. coli *chromosomal sequences from the dataset. In a final quality screening step, sequences with lengths below a certain threshold (150 bp), sequences with more than 4% ambiguous base calls (Ns), and sequences with a complexity below a given threshold (defined as sequence composed of more than 60% of a given nucleotide) were rejected. The rejected sequences were not used in unigene builds, but were retained in the database along with information as to why they were rejected.

### Unigene building

Unigene sets were built for each species by combining the sequences from all available libraries for that species. The sequences were first pre-clustered, and these clusters were then assembled using the cap3 [60] program. The cap3 identify parameter was set at 95%. Unigene sequences were also checked for length, complexity and contamination, and chimera detection was performed. The builds were uploaded to the PGN database, where each unigene was assigned a unique unigene ID. Subsequent unigene builds of the same libraries attributed new IDs to all unigenes. Unigenes from a newer build were then tracked to the older builds through the ESTs that they share, and as such, a complete history of unigene IDs is available on the website for tracking unigenes through the different builds.

### Annotation of sequence data

Several strategies were used to get the best possible annotation for the unigene sequences. First, BLAST was used to find the best match for each unigene sequence in the GenBank NR database, and in the complete coding sequences from *Arabidopsis *[34]. These annotations were stored in the database and serve as the primary source of FGP sequence annotation. To get a better overview for the annotations, we used the Gene Ontology (GO) system [38,61] to compare the sequence annotations from different species. Gene Ontology is a hierarchical system representing biological knowledge. Many model systems, such as *Arabidopsis*, are being annotated using GO [38,61]. The *Arabidopsis *GO annotations were transferred to each unigene that had a match with an *Arabidopsis *sequence with an e value less than 10^-20^. For the comparison of annotations among species, we focused on the biological functional GO category. We selected a number of high-level GO function terms as a GO slim vocabulary, and then mapped the GO annotations to the GO slim terms using the map2slim.pl script provided by the Gene Ontology consortium [38].

### Phylogenetic analyses of putative gene families

Floral Genome Project unigene assemblies and finished cDNAs were regularly subjected to phylogenetically-based classification. The general procedure was as follows: (1) for each PlantTribe of interest, use all *Arabidopsis*, rice and FGP sequences to search public databases [30,31,61] for similar protein coding genes (e < 10^-20^), (2) machine align all sequences using ClustalW [64], T-Coffee [65] or Muscle [66], (3) manually assess alignment, removing all highly divergent sequences, and then repeat step 2, (4) subject alignment to fast parsimony analysis both with and without branch support estimation [67,66,69]. The parsimony phylogeny shown for the DUF642 gene family (Fig. 4) is the strict consensus of 67 equally parsimonious trees estimated for an amino-acid alignment. The parsimony analysis was executed in PAUP* [66] with 100 random addition replicates and tree bisection-reconnection (TBR) branch swapping. Bootstrap analysis was performed with 250 replicates. The alignment is available in NEXUS format through the FGP website [29]. Two regions of the alignment were deemed questionable and removed from the phylogenetic analysis.

## Authors' contributions

VAA, DES, LAM, and JHL-M drafted the manuscript. JEC, WGF, LLL, YH, MB, SK, M-JY, BJB, and XZ acquired most of the data. RP-T and SES made essential contributions to Cucumis and Liriodendron EST library construction. PKW, DCI, TMS, LAM, and JHL-M designed and performed bioinformatic analyses. VAA, DES, JEC, MWF, ST, DGO, PSS, HM, CWD, and JHL-M conceived of the study, participated in its design and coordination, and helped complete the manuscript. All authors read and approved the final manuscript text.

## Figures and Tables

**Table 1:** Current (12/01/04) statistics for Floral Genome Project cDNA libraries, EST sequences and unigene builds. We will perform 10,000 EST sequencing reactions for each taxon listed in the top portion of the table and 2000 ESTs for each taxon in the bottom portion of the table.

**Taxon**	**Primary Titre (pfu)**	**Amplified Titre (pfu/ml)**	**Avg Insert size (bp)**	**ESTs to date**	**^a^Unigenes**	**^b^Observed Redundancy**
**Libraries for deep sequencing**						
*Welwitschia mirabilis *(m) – Gymnosperm	3.25E+05	6.00E+08	1382	3732	2771	34.7%
*Amborella trichopoda *(m) – Basal angiosperm	2.24E+06	1.37E+10	1611	4047	6099	41.5%
*Amborella trichopoda *(f)	4.98E+06	1.40E+10	1031	4442		
Nuphar advena (*Water lily, Spadderdock*) – *Basal angiosperm*	2.00E+06	3.20E+10	1134	8442	6205	36.1%
*Acorus americanus *(Sweet flag) – Basal monocot.	2.80E+06	6.00E+09	1083	5883	3067	28.8%
^*c*^*Asparagus officinalis *(m) – Transformable nongrass monocot	1.50E+06	1.20E+10	1468	5188	4560	61.5%
*Asparagus officinalis *(f)	1.30E+06	1.40E+10	1200	2174		
*Persea americana *(Avocado) – Cultivated magnoliid	2.74E+06	2.57E+10	1349	8735	5314	41.3%
*Liriodendron tulipifera *(Tulip Poplar, Yellow Poplar) – Transformable lumber species	3.00E+06	2.00E+10	1346	9531	6520	46.2%
*Saruma henryi *(Upright Wild Ginger) – member of Aristolochiaceae with bipartite perianth	1.97E+06	1.67E+10	1587	3230	2631	21.8%
*Eschscholzia californica *(California poppy) – Transformable basal eudicot	7.00E+06	1.68E+11	1702	9079	6015	46.18%
**Libraries for shallow sequencing**						
^*d*^*Cucumis sativus (m) *(Cucumber) – rosid	------	------	------	1107	1648	23.5%
^*d*^*Cucumis sativus (f)*	------	------	------	928		
Ribes americanum *(Black currant)*	2.58E+06	2.25E+10	1200	2238	1791	25.0%
^*e*^Vaccinium corymbosum *(Blueberry) *– *basal asterid*	------	------	------	1758	1549	13.5%
			**Total:**	70,514	48,170	

m indicates library constructed from male tissues; f for female tissues.

^*a*^Unigene numbers are shown in the first line for taxa with multiple libraries.

^b^Observed redundancy was measured for each taxon as (EST# – Unigene #)/Unigene#.

^c^5188 ESTs from the male *Asparagus *library were sequenced in collaboration with Mike Havey (University of Wisconsin) and Chris Town (TIGR).

^d^Male and female *Cucumis *flower bud libraries described by Perl-Treves et al [71]

^e^*Vaccinium *young inflorescence library was provided by Jeannine Rowland (USDA) [72].

**Table 2:** Number of FGP unigenes that are best BLAST hits to *Arabidopsis *floral developmental regulation genes, with corresponding tribe ID number and number of *Arabidopsis *and rice genes in these tribes. Species IDs: Aam, *Acorus americanus*; Aof, *Asparagus officinalis*; Ath, *Arabidopsis thaliana*; Atr, *Amborella trichopoda*; Eca, *Eschscholzia californica*; Ltu, *Liriodendron tulipifera*; Nad, *Nuphar advena*; Osa, *Oryza savita*; Pam, *Persea americanus*; She, *Saruma henryi*; Wmi, *Welwitschia mirabilis*.

**Gene ID**	**Annotation**	**Tribe^a^**	**Ath^b^**	**Osa^b^**	**Aam**	**Aof**	**Atr**	**Eca**	**Ltu**	**Nad**	**Pam**	**She**	**Wmi**	**Tot**
At2g45190	AFO, YABBY1	1010	4	7	3	1	1			3	2			12
At4g18960	AG, AGAMOUS	65	46	51			2	1			1			4
At4g09960	AGL11, MADS-box protein	65	46	51	1									1
At2g45660	AGL20, SOC	65	46	51							1		1	2
At4g24540	AGL24, MADS-box protein	65	46	51										
At2g03710	AGL3, MADS-box protein	65	46	51	1					1				2
At2g45650	AGL6, MADS-box protein	65	46	51	1		1	1	2	1			2	9
At4g37750	ANT, AINTEGUMENTA	123	18	38				2	1	1				4
At1g69120	AP1, APETALA 1	65	46	51						1	1			3
At4g36920	AP2, APELATA 2 (FL1, FLOWER1)	123	18	38			1		1	1	3			8
At3g54340	AP3, APETALA 3	65	46	51			1	2	1	2	2	1		9
At1g75950	ASK1	122	19	38			1		1	1				3
At5g42190	ASK2	122	19	38	3				2		7		1	16
At4g02570	AXR6, AUXIN RESISTANT 6	324	10	16	2		2	1	7	1	2	1		17
At1g01040	CAF, CARPEL FACTORY (SUS1)	446	8	13	2					1				3
At1g26310	CAL, CAULIFLOWER	65	46	51			1							1
At1g75820	CLV1, CLAVATA 1 (FASCIATA 3)	8	194	478				2						2
At1g65380	CLV2, CLAVATA 2	8	194	478			1							1
At2g27250	CLV3, CLAVATA 3	10933	1	0										
At1g69180	CRC, CRABS CLAW	1010	4	7										
At3g61850	DAG1, DOF AFFECTING GERMINATION 1	93	36	36							1			1
At2g33860	ETT, ETTIN	117	26	34		1								3
At3g59380	FTA, FARNESYLTRANSFERASE A	2266	1	5				1						1
At3g30260	FUL, FRUITFULL (AGL8)	65	46	51										
At4g20910	HEN1, HUA ENHANCER 1	3162	2	2	1				2					3
At2g06990	HEN2, HUA ENHANCER 2	1435	4	4	1		1				1	1		4
At5g64390	HEN4, HUA ENHANCER 4	601	10	7			2	1	1				1	6
At3g12680	HUA1, ENHANCER OF AG-4 1	469	10	9		1	2	1						4
At5g23150	HUA2, ENHANCER OF AG-4 2	1582	4	4										
At1g23420	INO, INNER NO OUTER	1010	4	7										
At5g16560	KAN, KANADI	100	25	43						1				1
At5g61850	LFY, LEAFY	7107	1	1										
At4g32551	LUG, LEUNIG	1572	2	6	1		1			1	2	1	1	7
At4g10350	NAM, NO APICAL MERISTEM	30	82	105										
At1g69490	NAP, NAC-LIKE, ACTIVATED BY AP3/PI	30	82	105			1							1
At1g68640	PAN, PERIANTHIA	253	10	21					1					1
At5g20240	PI, PISTILLATA	65	46	51				1		1	2		1	5
At2g34650	PID, PINOID	87	36	42				1		1				3
At2g28610	PRS, PRESSED FLOWER	626	8	8										
At5g35770	SAP, STERILE APETALA	9932	1	0										
At5g15800	SEP1, SEPALLATA1 (AGL2)	65	46	51	3		4	1		1				9
At3g02310	SEP2, SEPALLATA2 (AGL4)	65	46	51		1	1			1				3
At1g24260	SEP3, SEPALLATA3 (AGL9)	65	46	51				3	3		1			7
At1g43850	SEU, SEUSS	1762	4	3				2						2
At3g58780	SHP1, SHATTERPROOF 1 (AGL1)	65	46	51				1						1
At2g42830	SHP2, SHATTERPROOF 2 (AGL5)	65	46	51			1			2				3
At1g02065	SPL8, SQUAMOSA PROMOTER BINDING PROTEIN-LIKE 8	284	15	13	1				2	1				5
At3g23130	SUP, SUPERMAN	1084	5	5										
At5g03840	TFL1 TERMINAL FLOWER 1	397	6	17										
At3g22780	TSO1,	1120	4	6	1						1			2
At1g30950	UFO, UNUSUAL FLORAL ORGANS	4059	1	3										
At2g17950	WUS, WUSCHEL	626	8	8										
At4g00180	YABBY3	1010	4	7			1	1	2	1			1	6

^a^Tribe ID's are reference numbers for the PlantTribes database [33].

^b^Gene family size is represented by the number of rice (osa) and *Arabidopsis *(ath) genes in each medium stringency tribe.

**Table 3:** Number of FGP unigenes that are best BLAST hits to *Arabidopsis *flowering time genes, with corresponding tribe ID number and number of *Arabidopsis *and rice genes in these tribes (species abbreviations as in Table 2)

**Gene ID**	**Annotation**	**Tribe**	**Ath**	**Osa**	**Aam**	**Aof**	**Atr**	**Eca**	**Ltu**	**Nad**	**Pam**	**She**	**Wmi**	**Tot**
At2g45660	AGL20, SOC	65	46	51							1		1	2
At4g24540	AGL24, MADS-box protein	65	46	51										
At2g46830	CCA1, CIRCADIAN CLOCK ASSOCIATED 1	3546	2	1				1						2
At2g25920	ELF3	8701	1	1										
At5g11530	EMF1, EMBRYONIC FLOWER 1	10070	1	0										
At5g51230	EMF2, EMBRYONIC FLOWER 2	1026	5	6	1			3						4
At4g15880	ESD4, EARLY IN SHORT DAYS 4	10325	1	0										
At4g16280	FCA, FCA	2386	2	0										
At4g35900	FD, FD	6153	2	0										
At1g04400	FHA (CYR2, CRYPTOCHROME 2)	2549	2	3								1		1
At1g68050	FKF1, FLAVIN-BINDING KELCH DOMAIN F BOX PROTEIN	1047	6	5						1				1
At5g10140	FLC, FLOWERING LOCUS F	65	46	51										
At2g43410	FPA, FPA	2343	3	3				2						2
At5g24860	FPF1, FLOWERING PROMOTING FACTOR 1	311	11	16					1					1
At4g00650	FRI, FRIGIDA	6545	1	1					1					1
At1g65480	FT FLOWERING LOCUS T	397	6	17										
At3g59380	FTA, FARNESYLTRANSFERASE A	2266	1	5				1						1
At3g30260	FUL, FRUITFULL, AGL8	65	46	51										
At4g25530	FWA, FWA	230	18	15										
At5g13480	FY, FY	7822	1	1			2							2
At1g14920	GAI, GA INSENSITIVE	74	27	62										
At1g22770	GI, GIGANTEA	8967	1	1		1	3		2		2	1		10
At4g08920	HY4, ELONGATED HYPOCOTYL 4 (CRY1)	2549	2	3			1				3			5
At2g23380	ICU1, INCURVATA 1	2735	3	2										
At4g02560	LD, LUMINIDEPENDENS	8840	1	1			2							2
At5g61850	LFY, LEAFY	7107	1	1										
At1g01060	LHY, LATE ELONGATED HYPOCOTYL	3546	2	1	1					1				2
At1g77080	MAF1, MADS AFFECTING FLOWERING 1	65	46	51										
At5g65050	MAF2.4, MADS AFFECTING FLOWERING2	65	46	51										
At5g65060	MAF3, MADS AFFECTING FLOWERING 3	65	46	51										
At5g65070	MAF4.5, MADS AFFECTING FLOWERING 4 VARIANT V	65	46	51										
At5g65080	MAF5.2, MADS AFFECTING FLOWERING 5 VARIANT II	65	46	51										
At2g19520	NFC4, FVE	1299	6	3	2		1	1	1	3				8
At1g09570	PHYA, FAR RED ELONGATED 1	1254	5	4				2	1	3	1			7
At2g18790	PHYB, PHYTOCHROME B	1254	5	4	1				1		2	1		5
At5g35840	PHYC, PHYTOCHROME DEFECTIVE C	1254	5	4			1		1					2
At4g16250	PHYD, PHYTOCHROME DEFECTIVE D	1254	5	4										
At4g18130	PHYE, PHYTOCHROME DEFECTIVE E	1254	5	4										
At1g73590	PIN1, PIN-FORMED 1	405	8	14							2			2
At2g01570	RGA1, REPRESSOR OF GA1-3 1	74	27	62			1							1
At1g02065	SPL8, SQUAMOSA PROMOTER BINDING PROTEIN-LIKE 8	284	15	13	1				2	1				5
At3g11540	SPY, SPINDLY	6916	1	1				1		1				2
At2g22540	SVP, SHORT VEGETATIVE PHASE	65	46	51	1		1				1			3
At2g28290	SYD, SPLAYED	135	20	30							1			1
At2g26670	TED4, ELONGATED HYPOCOTYL 1	2358	4	2		1								1
At5g03840	TFL1, TERMINAL FLOWER 1	397	6	17										
At5g17690	TFL2,	5181	1	2					1		1		1	4
At5g61380	TOC1, PSEUDO-RESPONSE REGULATOR 1	769	6	8					1	2			2	5
At5g61150	VIP4, VERNALIZATION INDEPENDENCE 4	2513	1	4				2						2
At3g18990	VRN1, REDUCED VERNALIZATION RESPONSE 1	4792	2	1	1									1
At4g16845	VRN2, REDUCED VERNALIZATION RESPONSE 2	1026	5	6										
At1g80730	ZFP1, ZFP1	511	6	13										
At5g57360	ZTL, ZEITLUPE	1047						2	1					3

**Table 4:** Distribution of best matches to floral organ-specific *Arabidopsis *genes [40] among seven unigene sets (species abbreviations as in Table 2). Shown in bold are two DUF642-domain genes with differential expression in petals.

**Organ**	**Gene ID**	**Annotation**	**Tribe**	**Ath**	**Osa**	**Aam**	**Aof**	**Atr**	**Eca**	**Ltu**	**Nad**	**Pam**	**She**	**Wmi**	**Tot**
carpel	At5g44635	similar to putative CDC21 protein	549	8	10				1		1	1			3
carpel	At1g71691	hypothetical protein with GDSL-like motif	40	84	79	1			1	1		2	1		6
carpel	At5g53120	spermidine synthase	2331	3	3			1		1	3	1			6
carpel	At5g07280	receptor-like protein kinase-like protein	8	194	478	2	1		2					1	7
carpel	At3g51860	Ca2+/H+-exchanging protein-like	823	6	7		1	1		1	2	2	1		8
carpel	At5g06860	polygalacturonase inhibiting protein (PGIP1)	8	194	478	1	1		1	1	1				8
carpel	At5g02540	putative protein	348	11	14	1		1	1	1	1	1	2	1	9
carpel	At5g59320	nonspecific lipid-transfer protein precursor – like	277	11	18		1	1	1	2	2	2		1	10
petal	At3g62700	glutathione-conjugate transporter, putative	192	16	22	1		1	2	1					5
**petal**	**At5g11420**	**putative protein (DUF642 Domain)**	**328**	**10**	**16**		**1**	**1**	**2**	**1**	**1**				**6**
**petal**	**At1g80240**	**putative protein (DUF642 Domain)**	**328**	**10**	**16**		**1**	**1**	**2**	**1**	**1**				**6**
sepal	At1g69120	floral homeotic gene APETALA1	65	46	51						1	1			3
stamen	At5g14780	formate dehydrogenase (FDH)	4097	1	3		1	1		1	1	3	1		8
stamen	At1g52570	phospholipase D, putative	300	10	17			4		1	1	2	1		9
stamen	At3g09390	metallothionein-like protein	6057	2	0			1		2	1	2	2		10
stamen	At3g03080	putative NADP-dependent oxidoreductase	315	14	13	1		2	1	1	1	3			11
stamen	At3g62290	ADP-ribosylation factor-like protein	197	19	17	4	3	2	1	1		2		1	14
stamen	At5g43330	cytosolic malate dehydrogenase	1443	4	4	3	2	1	1	3	1	3			14
stamen	At1g13950	initiation factor 5A-4	1208	3	7		1	3	1	3	2	5			18
stamen	At5g45775	Expressed protein	923	4	7	2		1	7	2	1	3	1	1	18
stamen	At5g14670	ADP-ribosylation factor – like protein	197	19	17	3		2	6	3	3	4		2	24
